# Axonal maintenance, glia, exosomes, and heat shock proteins

**DOI:** 10.12688/f1000research.7247.1

**Published:** 2016-02-22

**Authors:** Michael Tytell, Raymond J. Lasek, Harold Gainer

**Affiliations:** 1 Department of Neurobiology and Anatomy, Wake Forest University School of Medicine, Winston-Salem, NC, 27157, USA; 2 Department of Anatomy, Case Western Reserve University School of Medicine, Cleveland, OH, 44106, USA; 3 Laboratory of Neurochemistry, National Institute of Neurological Disorders and Stroke, National Institutes of Health, Bethesda, 20892, USA; 4 Marine Biological Laboratory, Marine Biological Laboratory, Woods Hole, MA, 02543, USA

**Keywords:** Axonal maintenance, glia, exosomes, heat shock proteins, HSP, axoplasm

## Abstract

Of all cellular specializations, the axon is especially distinctive because it is a narrow cylinder of specialized cytoplasm called axoplasm with a length that may be orders of magnitude greater than the diameter of the cell body from which it originates. Thus, the volume of axoplasm can be much greater than the cytoplasm in the cell body. This fact raises a logistical problem with regard to axonal maintenance. Many of the components of axoplasm, such as soluble proteins and cytoskeleton, are slowly transported, taking weeks to months to travel the length of axons longer than a few millimeters after being synthesized in the cell body. Furthermore, this slow rate of supply suggests that the axon itself might not have the capacity to respond fast enough to compensate for damage to transported macromolecules. Such damage is likely in view of the mechanical fragility of an axon, especially those innervating the limbs, as rapid limb motion with high impact, like running, subjects the axons in the limbs to considerable mechanical force. Some researchers have suggested that local, intra-axonal protein synthesis is the answer to this problem. However, the translational state of axonal RNAs remains controversial. We suggest that glial cells, which envelop all axons, whether myelinated or not, are the local sources of replacement and repair macromolecules for long axons. The plausibility of this hypothesis is reinforced by reviewing several decades of work on glia-axon macromolecular transfer, together with recent investigations of exosomes and other extracellular vesicles, as vehicles for the transmission of membrane and cytoplasmic components from one cell to another.

## Introduction

The axon is a unique cellular structure. It extends from the neuron’s cell body as a cylindrical process with a constant, stable diameter and length, differing from all other cellular extensions (including dendrites) that are typically tapered and dynamic in shape ^1, 2^. In addition, axons extend for very long distances, often many orders of magnitude greater than the cell body’s diameter and volume. Maintenance of this stable structure is critical for the principal function of the axon, i.e. to conduct the action potential to its synaptic terminal with a determined conduction velocity. How this seemingly fragile cylinder is maintained for the lifespan of an organism, especially in large ones, has puzzled researchers for decades. We undertook this review to integrate historical and contemporary research findings that suggest a mechanism for the maintenance of macromolecules in the mature axon that are critical for its structure and function.

The traditional view is that the macromolecules (e.g. proteins) that are important for axonal function are synthesized in the neuronal cell body and supplied to the axon by axonal transport mechanisms ^2– 7^. The adequacy of this mechanism to compensate for the turnover of proteins in the axon has been challenged ^8^, and it has been posited by several groups that “local” protein synthesis in the axon itself may be needed to supplement the source from the neuronal soma ^8– 11^. However, we propose here that a more likely alternative supplementary mechanism is the transfer of macromolecules from the adaxonal glia to the axon by extracellular vesicles (EVs), e.g. exosomes. The idea of glia supplying proteins to vertebrate axons was first proposed by Marcus Singer ^12– 14^, who showed by insightful interpretation of autoradiographic results that newly synthesized Schwann cell proteins that had incorporated radioactive amino acids were transferred into the peripheral axons that they ensheathed.

Our thesis here is that glia to neuron transfer of macromolecules is likely to occur via EVs, such as exosomes. We summarize recent advances in exosome research that indicate that exosomes are the primary vehicles for intercellular macromolecular transfer. Additionally, we describe evidence for a unifying principle that a key function of glia-neuron transfer is neuroprotection by heat shock protein (Hsp)-containing exosomes.

## Lessons from invertebrate axons

### The squid giant axon

Giant axons in invertebrates offer a unique opportunity to study the possibility of *de novo* protein synthesis in axoplasm because pure axoplasm can be collected for physiological and biochemical analyses. The first biochemical experiments that used the squid giant axon isolated from its cell bodies to determine whether axonal protein synthesis occurred were performed by Giuditta and colleagues ^15^. These authors observed the appearance of radioactively labeled proteins in extruded axoplasm and noted that there could be two potential sources of the newly synthesized axonal proteins, intra-axonal protein synthesis or the surrounding glia called Schwann cells. The next important investigations showed that isolated axoplasm from squid and marine worm ( *Myxicola*) giant axons contained transfer RNA, but not detectable ribosomal RNA (rRNA) ^16^, and thus seemed to discount the possibility of *de novo* protein synthesis in the axoplasm. In this regard, it should be noted that Giuditta and colleagues continue to report finding various components of protein translational machinery in isolated squid axoplasm ^9, 17– 21^, including rRNA ^22^ and ribosomes ^23^, as support for the concept that *de novo* protein synthesis could be occurring in the axoplasmic compartment ^24^.

An alternative proposal about the source of radiolabeled proteins in squid giant axon axoplasm was presented as the “Glia-Neuron Protein Transfer Hypothesis” published in 1977. These publications posited that the source of the newly synthesized radiolabeled proteins found in the axoplasm was the adaxonal Schwann cell sheath ^25, 26^. This hypothesis was based on the facts that (1) the giant axon did not contain a significant amount of ribosomes or rRNA; (2) isolated axoplasm was unable to synthesize labeled proteins from radioactive amino acids; (3) injection of RNase into the giant axon did not reduce the appearance of newly synthesized proteins in the axoplasm of the isolated giant axon ^26^; and (4) incubation of the squid giant axon in ^3^H-leucine while it was cannulated and perfused with an artificial axoplasm solution demonstrated the appearance of newly synthesized proteins in the perfusate, continuously for over 8 hours of incubation, whether or not RNase was included in the perfusion solution. However, when puromycin was included in the extra-axonal incubation solution to block *de novo* protein synthesis in the glial sheath, it completely prevented the appearance of labeled proteins in the perfusate ^25^. Taken together, these data strongly indicate that the squid giant axon cannot synthesize proteins *de novo* and that the most likely source of the newly synthesized proteins is its adaxonal Schwann cell sheath.

Later studies by Lasek and Tytell in the squid giant axon strengthened and extended the “Glia-Neuron Protein Transfer Hypothesis”, and focused on the nature of the transported proteins and the possible mechanisms of the intercellular transfer. In one study, the labeled proteins in the axoplasm, the glial sheath containing the Schwann cells, and the stellate ganglion containing the cell bodies of the giant axon were analyzed separately and compared ^27^. Over 80 glial polypeptides were found to be selectively transferred into the axoplasm and many of these were distinct from groups of stellate ganglion proteins, which were presumed to include those destined for the axon via axonal transport. Three of the more highly labeled transferred glial polypeptides were actin, a fodrin-like polypeptide, and a 70–80 kDa polypeptide they named traversin. Traversin was later identified as being an Hsp, since its expression and transfer was increased by exposure of the axon to elevated temperatures, as was another prominent 95 kDa protein ^28^. Since both the 70 kDa and 95 kDa proteins were similar in molecular weight and charge to Hsps described in other systems, this suggested that they were members of the Hsp family and that the glia provided the axon with proteins that may be involved in the reaction to metabolic stress. A follow-up study used a fluorescent vesicular reporter, acridine orange, that selectively stained acidic vesicular structures in glial cells in the giant axon sheath. The results suggested that some of those fluorescent vesicles in the glia were transferred into the axoplasm ^29^. Several speculations for the mechanisms that could produce these transfers were considered. These were glial exocytosis coupled with neuronal endocytosis, diffusion through intercellular channels, modified phagocytosis, and protein translocation analogous to the transfer of newly translated proteins into the rough endoplasmic reticulum (RER) ^30, 31^.

### Crayfish motor and giant axons

In 1967, Hoy, Bittner, and Kennedy ^32^ made a remarkable observation during studies on axonal regeneration in the crayfish claw. These authors found that after transection of the motor nerves innervating the muscles in the crayfish claw, stimulation of the distal stumps of these nerves could evoke normal synaptic potentials in the muscle more than 100 days after transection ^32^. This functional survival of the crayfish motor axon after transection suggested that the adaxonal glia might contribute to its long-term survival.

Subsequent experiments by Sarne *et al.* ^33, 34^ found that in the transected crayfish nerve the small sensory (cholinergic) axons degenerated, whereas the non-cholinergic motor axons did not. The motor axons were associated with a robust glial sheath, whereas the sensory axons had relatively poor glial coverage, often with many axons sharing a single glial sheath. Incubation of the isolated nerve with radioactive amino acids produced an autoradiographic picture similar to that seen in the squid giant axon experiments, and transection of the axon seemed to produce an increase in the amount of protein synthesized ^33^. Since the neurotransmitters in the sensory axons (acetylcholine) and in the inhibitory motor axons (γ-aminobutyric acid, GABA) were known, Sarne *et al.* ^34^ studied the effects of transection of these axons from their cell bodies on the contents of their respective synthetic enzymes, choline acetyltransferase (CAT) in the sensory axons and glutamic acid decarboxylase (GAD) in the motor neurons. Transection of the axons *in vivo* produced a dramatic decrease in CAT activity, presumably due to the degeneration of the cholinergic axons (see also 35), but the GAD activity remained unchanged even 14 days after transection. Complete isolation of the axons in organ culture also did not alter the GAD activity in the tissue. However, incubation of the cultured tissue in the eukaryotic protein synthesis inhibitor anisomycin caused a complete loss of GAD activity. Therefore, it appeared that a local synthesis in the nerve fibers (presumably in the glia surrounding the inhibitory motor axon) was responsible for the maintenance of the GAD activity.

Similar findings were made by Meyer and Bittner ^36, 37^ on the medial giant axon (MGA) in the central nervous system of the crayfish, which showed that the kinetic appearance of the autoradiographic grains representing newly synthesized proteins, first in the glia and later in the axon, were consistent with a transfer of glial proteins to the axon. Bittner and colleagues have done extensive work since then studying the long-term survival of axons in the absence of cell bodies in the central nervous system of crayfish. The survival of the MGA in the crayfish nerve cord was found to be particularly dependent on intercellular transfer of macromolecules from surrounding glia ^35, 38^. Of particular interest here is the identification of Hsps of the 70 kDa family as prominent proteins found in the crayfish MGA ^39^. The Hsp70 proteins represented 1–3% of the total protein in the axoplasm of the MGAs. After heat-shock treatment, overall protein synthesis in the glial sheath was decreased compared with that of control axons, but newly synthesized proteins of 72 kDa, 84 kDa, and 87 kDa appeared in both the axoplasm and the sheath and were interpreted as being the stress-inducible Hsps. These authors proposed that the Hsps in the MGAs may help these axons maintain essential structures and functions following acute heat shock.

## The significance of intra-axonal RNAs

The above work clearly showed that glia-axon protein transfer occurs in many but not all axons and is particularly important for the maintenance of large invertebrate axons. This is also true for many mature vertebrate axons ^12, 40^. However, there have been recent reports of the presence of intra-axonal RNAs shown to be derived from either axonal transport or glia-axon transfer mechanisms ^10, 41– 43^. This has led many investigators to infer that the presence of intra-axonal RNAs represents evidence for intra-axonal protein synthesis ^10, 41, 42, 44^. There is also excellent evidence presented that *de novo* protein synthesis can occur in neuronal compartments other than the cell body, e.g. in dendrites ^45– 48^, in growth cones during development and regeneration, and in immature axons in culture ^10, 21, 42, 44, 49^. Nevertheless, the presence of mRNAs and other translational machinery in mature axons should not be taken as evidence of *de novo* protein synthesis in that structure. For example, squid giant axoplasm contains considerable neurofilament protein mRNAs ^50^ but does not synthesize neurofilament proteins (see 26 and Gainer [in preparation]). Another example that the presence of mRNAs does not imply protein synthesis comes from studies of posterior pituitary axons and terminals. Even though these structures contain oxytocin and vasopressin precursor protein mRNAs transported from the hypothalamic magnocellular neurons and they increase in amount with functional activity ^51– 58^, they are not used to synthesize the precursor proteins as would occur on the RER in the neuronal cell bodies, probably because RER is absent from the pituitary axons and nerve endings ^59– 61^. The issue of whether the presence of intra-axonal RNAs is concordant with evidence of intra-axonal protein synthesis will depend on the development of new methods to visualize protein synthesis *in situ* ^62, 63^. Whether or not local protein synthesis occurs in the axon, in principle, the glia can provide additional proteins not synthesized by the neuron, such as Hsp70, that can protect axonal function in the face of physical trauma and metabolic stress.

## Glia-axon macromolecular transfer, extracellular vesicles, and Hsp70

In the 1980s, before exosomes and other types of EVs were recognized as vehicles for the movement of cytoplasmic constituents from one cell to another, it was common knowledge that certain cell types release material by the blebbing of small, membrane-bound packets from their apical surfaces, a process known as apocrine secretion first described in the middle 1800s by several biologists including Purkinje. Mammary gland secretory epithelium and apocrine glands in the skin are two examples of tissues that exhibit this type of secretion. Thus, this concept was familiar when a few reports began to appear in the 1980s that showed other cell types release cytoplasmic proteins during normal function. A now well-known example is in the development of reticulocytes into mature red blood cells (RBCs), which dispose of many of the intracellular and membrane components unneeded in mature RBCs by sequestering things like transferrin receptors and a variety of other cytoplasmic proteins into small vesicles within multivesicular bodies (MVBs) ^64– 66^. When the MVBs fused with the cell surface, the small vesicles were released ^66^. Interestingly, those EVs included Hsc70, the constitutively synthesized isoform of Hsp70 ^67^. That fact suggested to one of us (Tytell) that there might be some similarity between the microvesicle production in reticulocytes and the mechanism used by adaxonal glia in the squid axon to transfer proteins to the axon. It stimulated work reported a few years later that provided evidence that glial-derived vesicles were transferred into the axoplasm of the squid axon ^29^.

During the late 1980s and 1990s, work on membrane-bound vesicle release from cells increased greatly and the term exosome was applied more widely. However, released vesicles represent a very heterogeneous group, leading to attempts to classify them based on how they are produced, released, and isolated ^68^. As reviewed in Smith *et al.* ^69^, there are three groups of EVs in addition to the 30–100 nm vesicles commonly referred to as exosomes: (1) shedding vesicles that arise from blebbing from the cell surface as in apocrine secretion referred to earlier; (2) non-infectious retrovirus-like particles containing a subset of retroviral proteins; and (3) membrane-bound structures arising from apoptotic cells. Additional reviews by Théry ^70^ and Harding *et al.* ^64^ focusing on exosomes, showed that a huge variety of cytoplasmic and membrane components, including mRNAs and small, noncoding RNAs, could be released via exosomes from a variety of cells, and that the process of release is under the control of several of the Rab GTPases. This complexity has led to debates about the appropriate nomenclature for these structures, so we will use exclusively the acronym EVs to refer to them, as recommended by Gould and Raposo ^68^. The diverse group of molecules found in EVs are generated by many different cell types that contain a common set of molecules plus unique proteins that are associated with cell type-related functions ^71^. The common proteins include the tetraspanins, CD9 and CD63, that are involved in the biogenesis of exosomes and represent biomarkers for these organelles. Hence, they are often used for their selective isolation. Tetraspanins are a family of 30 proteins and specific members of this protein family can differ in exosomes that are derived from different tissues ^72^. They consist of four transmembrane domains that form a tertiary structure believed to cluster proteins required for intraluminal vesicle (ILV) formation, an intermediary step in EV generation. Several other proteins typically found in EVs include Tsg101, Alix, Rab-GTPases, and annexins ^71– 73^. The formation of EVs is a complex process, beyond the scope of this review, and excellent, detailed discussions about this process can be found elsewhere ^70, 71, 74– 77^. In brief, EVs may be derived from the cell’s endosomal network that sorts ILVs into late endosomes, also referred to as MVBs. The sorting machinery involves an endosomal component known as the endosomal sorting complex (ESCRT), responsible for various aspects of the transport and sorting of the exosomal cargo ^76^. The MVBs containing ILVs fuse with the plasma membrane and release the exosomes into the extracellular space where they can interact with various target tissues.

Although the study of endosomal functions in the nervous system is still nascent, there is considerable evidence that EVs play significant roles as vehicles for communication in both directions between neurons and glia ^78^. Both of these cells secrete exosomes and each cell type in the nervous system produces distinctive exosomes ^78^. For example, astrocyte-derived exosomes contain Hsp/Hsc70, various growth factors such as fibroblast growth factor (FGF)-2 and vascular endothelial growth factor (VEGF), and angiogenic factors. Oligodendroglial exosomes contain various myelin proteins such as 2',3'-cyclic nucleotide 3'-phosphodiesterase (CNP) and proteolipid protein (PLP), Hsp-70, -71, -90, factors that inhibit myelin formation, trophic factors for axonal integrity, various Rab proteins, and small RNAs. Microglial exosomes contain proinflammatory cytokines, interleukin-1beta, P2X7 receptors, major histocompatibility complex (MHC) class II, and various degradative enzymes ^75, 78, 79^. In addition to the sorting and secretion of normal proteins to glial exosomes, they can accumulate and secrete abnormal molecules under pathological circumstances, serving as “double-edged swords” in being propagators of neurodegenerative disease and serving as biomarkers for medical diagnoses ^69, 75, 78, 80, 81^. Recently, this pathological function has been a research focus in cancer ^82– 86^, as well as in the nervous system in glioblastoma ^69, 87– 89^. Prion propagation has also been attributed, in part, to exosomes ^90, 91^.

Thus, exosome release represents a still-unfolding story of cell-to-cell communication with potentially widespread effects on target cells. Especially relevant to this review is the demonstration by Lancaster and Febbraio ^92, 93^ that it was via exosomes that Hsp70 was released from a variety of cultured cell types, including human peripheral blood monocytes. They showed that heat stress of 1 hour at 43°C increased significantly the Hsp70 content of those exosomes, although it did not alter the rate of exosomal release. That observation suggested that Hsp70 content of exosomes reflects the relative abundance of the protein in the cell cytoplasm. Whether Hsp70 may be specifically directed to exosomes in stressed cells remains unknown.

## Neuroprotective functions of exosomes and Hsps

Many neurons, especially those with axons that are hundreds to thousands of times the diameter of the soma of origin (that is, centimeters or more in length), lack the typical stress response seen in most other cells. They do not increase their synthesis of Hsps in response to the level of metabolic stress typically encountered in an organism, such as fever-level hyperthermia (typically up to 45°C) or hypoxia ^94, 95^. This aberrant stress response has been considered paradoxical, as the vulnerability of neurons to such stresses is well known. Recently, differential regulation between glia and neurons of the heat shock factor, HSF1, binding to the gene promoter, was shown to underlie this distinction ^96^. Glial and other non-neuronal cells like ependymal cells, however, have the typical stress response, increasing the synthesis of Hsp70 and some of the other Hsps markedly ^94, 95, 97– 101^. Nonetheless, neurons do become more stress resistant despite this deficient stress response if Hsp70 is present in extracellular fluid (reviewed in 102; see also 97, 103, 104). The fact that soluble Hsp70 has a domain that allows it to interact with and pass through the cell membrane may be another way that glia contribute to neuronal acquisition of stress tolerance ^105^. However, because it seems likely that exosomes are the predominant means by which glia and other cells release their Hsp70, we suggest that the uptake of glial EVs containing Hsp70 is the likely way that neurons benefit from the robust glial stress response. In fact, several other reviews and reports ^78, 99, 106, 107^ strongly support this hypothesis and provide evidence for glial EVs supplying a variety of molecules promoting neuron function even under nonstressful conditions ^79, 100, 107, 108^.

## Conclusions and future directions

**Figure 1.**
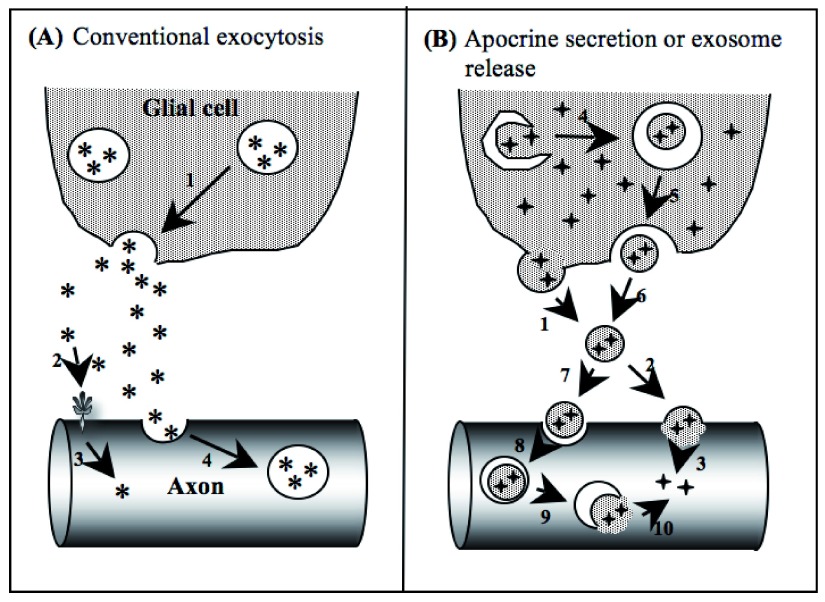
(from Tytell *et al.* ^102^). Potential mechanisms for the glial release and axonal uptake of heat shock proteins (Hsps). **Panel A**: An unknown mechanism allows Hsp70 (the stress-inducible form) or Hsc70 (the constitutively synthesized form), represented by asterisks, to become concentrated in secretory vesicles (hereafter, the proteins will be jointly designated as Hsp/c70). Arrow 1 indicates conventional exocytosis of Hsp/c70 into the extracellular space. Evidence for that process is limited ^110^. Arrow 2 indicates interaction of the extracellular Hsp/c70 with the axonal membrane, reflected by a change in its shape, followed by diffusion into the axoplasm, where it is free to interact with other cytoplasmic components. This possibility must be considered because Hsp/c70 is known to interact with membrane phospholipids (reviewed in De Maio *et al.* ^111^) and to include an amino acid sequence permitting passage through cell membranes ^105^). Arrow 4 indicates uptake of Hsp/c70 by conventional endocytosis, after which it is present in endocytotic vesicles. From there it may diffuse through the endosomal membrane to enter the cytoplasm or it may remain inside as the endosome cycles through the endolysosomal pathway of the neuron. How that process might affect stress tolerance is unknown. **Panel B**: Depiction of two ways that exosomes containing glial cytoplasmic Hsp/c70 (4-pointed stars) could gain access to the cytoplasm of the axon. On the left side, arrow 1 indicates conventional apocrine secretion, in which small vesicles containing a mixture of cytoplasmic constituents bud off the cell surface. Alternatively, some of the cytoplasm of the donor may be enclosed within a vesicle by the process of autophagy (arrow 4). The multivesicular body resulting from autophagy may then be released by the glial cell via exocytosis (arrows 5 and 6). Thus, both apocrine secretion and exosome release yield the same result, a membrane-enclosed vesicle containing cytoplasmic constituents in the extracellular space. The released vesicle then may interact with the axon in either of two hypothetical ways. It may fuse with the plasmalemma of the recipient cell, releasing its contents, including Hsp/c70, into the neuron’s cytoplasm (indicated by arrows 2 and 3). Alternatively, the released vesicle may be phagocytosed by the axon, forming another multivesicular body. Then, the inner vesicle membrane may fuse with the outer vesicle membrane in a process analogous to exocytosis, releasing the Hsp/c70 and other cytoplasmic constituents from the glial cell-derived vesicle into the cytoplasm of the axon (indicated by arrows 8–10).

Despite the explosion of work on EVs in the last few years, a fundamental understanding of how these and other microvesicles interact with target cells such as neurons has only recently begun to be elucidated. There are a number of possible mechanisms by which neurons and other target cells could internalize these small EVs. Frühbeis *et al.* ^78, 108^ have reported that at least one process involves dynamin-dependent endocytosis. Although that process would place the exosome’s contents within a membrane-bound organelle inside the cell and thus would make them inaccessible to the cytoplasm, they showed that some of the EV components were functional within the neurons in a way that required access to the cytoplasm. One such example is the acquisition of glial Hsp70 by neurons leading to increased neuronal stress tolerance ^108^. That suggests that the exosomal Hsp70 must gain access to the neuronal cytoplasmic compartment so that it can protect and refold cytoplasmic proteins in danger of denaturation and aggregation. Future research should focus on ways to follow exosomal contents so that we can understand how the exosomal cargo enters the target cell cytoplasm. As shown in Figure 1, we suggest several ways cytoplasmic localization of exosomal contents could happen: (1) by an intracellular membrane fusion event analogous to exocytosis of secretory vesicles; (2) by fusion of an exosome with the plasmalemma; or (3) by passage of exosomal molecules through membranes, either directly (as is possible for Hsp70 ^105^) or via carriers that are analogous to the way cytoplasmic proteins destined for the mitochondrial matrix are carried through the two mitochondrial membranes, a process that, coincidently, is dependent on Hsp70 ^109^.

Another intriguing area just beginning to be explored is to determine how to engineer exosomes and other EVs as therapeutic vehicles for targeted drug, gene, protein, or lipid delivery ^71, 73, 75, 81^. Recently, Yuyama *et al.* ^112^ have shown that neuronal exosomes that contained abundant glycosphingolipids could sequester intracerebral amyloid-β peptide in the brains of amyloid precursor protein transgenic mice and decrease amyloid-β and amyloid depositions in the brain. Thus, in addition to furthering our understanding of the long-standing puzzle of how glia support neurons and their axons, a better understanding of the function of exosomes and other EVs may provide new tools for combatting neurodegenerative diseases.
